# Radiographic sarcopenia predicts postoperative infectious complications in patients undergoing pancreaticoduodenectomy

**DOI:** 10.1186/s12893-017-0261-7

**Published:** 2017-05-26

**Authors:** Kosei Takagi, Ryuichi Yoshida, Takahito Yagi, Yuzo Umeda, Daisuke Nobuoka, Takashi Kuise, Toshiyoshi Fujiwara

**Affiliations:** Department of Gastroenterological Surgery, Okayama University Graduate School of Medicine, Dentistry, and Pharmaceutical Sciences, 2-5-1 Shikata-cho, Kita-ku, Okayama, 700-8558 Japan

**Keywords:** Sarcopenia, Complication, Infection, Pancreaticoduodenectomy

## Abstract

**Background:**

Recently, skeletal muscle depletion (sarcopenia) has been reported to influence postoperative outcomes after certain procedures. This study investigated the impact of sarcopenia on postoperative outcomes following pancreaticoduodenectomy (PD).

**Methods:**

We performed a retrospective study of consecutive patients (*n* = 219) who underwent PD at our institution between January 2007 and May 2013. Sarcopenia was evaluated using preoperative computed tomography. We evaluated postoperative outcomes and the influence of sarcopenia on short-term outcomes, especially infectious complications. Subsequently, multivariate analysis was used to assess the impact of prognostic factors (including sarcopenia) on postoperative infections.

**Results:**

The mortality, major complication, and infectious complication rates for all patients were 1.4%, 16.4%, and 47.0%, respectively. Fifty-five patients met the criteria for sarcopenia. Sarcopenia was significantly associated with a higher incidence of in-hospital mortality (*P* = 0.004) and infectious complications (*P* < 0.001). In multivariate analyses, sarcopenia (odds ratio = 3.43; *P* < 0.001), preoperative biliary drainage (odds ratio = 2.20; *P* = 0.014), blood loss (odds ratio = 1.92; *P* = 0.048), and soft pancreatic texture (odds ratio = 3.71; *P* < 0.001) were independent predictors of postoperative infections.

**Conclusions:**

Sarcopenia is an independent preoperative predictor of infectious complications after PD. Clinical assessment combined with sarcopenia may be helpful for understanding the risk of postoperative outcomes and determining perioperative management strategies.

## Background

Pancreaticoduodenectomy (PD) is one of the most complicated procedures in the field of gastroenterological surgery. As a result, the postoperative mortality and morbidity rates after PD remain high (2.8–3.5% and 40%, respectively) according to nationwide surveys performed in Japan [1, 2]. Furthermore, infectious complications after pancreatic surgery are common during the postoperative course, and can lead to fatal outcomes [3]. High morbidity rates are associated with the need for further treatment and extended hospital stays. Thus, a precise method of predicting postoperative complications is urgently required to ensure patient safety following PD.

Recent studies have shown that computed tomography (CT)-assessed sarcopenia (radiographic sarcopenia), which is characterized by skeletal muscle depletion and is an objective predictor of frailty, is associated with poor outcomes in gastrointestinal and hepatopancreatobiliary malignancies [4]. Previous studies have also shown that sarcopenia is associated with short-term outcomes, and especially with postoperative pancreatic fistula (POPF), in patients undergoing PD. [5–8] However, the influence of sarcopenia in terms of postoperative infectious complications has not been assessed in detail. We hypothesized that sarcopenia is associated with postoperative infections in patients undergoing PD.

With the above in mind, the aim of this retrospective study was to investigate postoperative outcomes following PD and to assess the influence of sarcopenia on short-term outcomes. In particular, we focused on the relationship between sarcopenia and infectious postoperative complications in patients following PD.

## Methods

### Patients

We retrospectively reviewed the medical records of 241 consecutive patients who underwent PD at the Okayama University Hospital between January 2007 and May 2013. This study was approved by the Ethics Committee of the Okayama University Graduate School of Medicine, Dentistry, and Pharmaceutical Sciences and Okayama University Hospital, and was conducted in accordance with the tenets of the Declaration of Helsinki. Due to the retrospective nature of the study, the need for informed consent was waived.

### Clinical data

For all enrolled patients, the following demographic and clinical data were evaluated as preoperative factors: sex, age, height, weight, body mass index (BMI), body surface area (BSA), American Society of Anesthesiologists (ASA) physical status, laboratory values (albumin level and total lymphocyte count), liver function according to the Child–Pugh score, comorbidities, etiology of disease, and preoperative biliary drainage. ASA physical status was preoperatively evaluated by an anesthesiologist. Preoperative biliary drainage included endoscopic biliary drainage and percutaneous transhepatic biliary drainage. Data regarding operative time, amount of blood loss, portal vein reconstruction, pancreatic texture (soft or hard) assessed by the surgeon intraoperatively, and main pancreatic duct diameter, were recorded as intraoperative factors.

### Surgical procedures and perioperative management

The standard surgical procedure was subtotal stomach-preserving PD. The basic reconstruction of the digestive system was performed by means of a modification of the method described by Child [9]. Pancreatojejunostomy was performed with a duct-to-mucosa anastomosis. Hepatojejunostomy was performed 10 cm distal to the pancreatojejunostomy. Gastrojejunostomy was performed by means of a two-layer anastomosis 50 cm distal to the hepatojejunostomy. A Braun anastomosis was also added. The details of these surgical techniques have been reported previously [10, 11]. In most cases, three drains were placed around the pancreatic and biliary anastomoses. All patients received prophylactic antibiotics every 3 h intraoperatively and for 3 d postoperatively.

Postoperative care was performed in a specialized surgical unit. Patients did not routinely receive somatostatin analogues and nutritional supplementation during the perioperative period. The drains were removed after postoperative day 5 if the drainage fluid was clear and no bacterial contamination was detected.

### Image analysis and definition of sarcopenia

Diagnostic CT images taken within 3 months prior to surgery were chosen and evaluated using a CT image analysis system (Synapse Vincent; Fujifilm Medical, Tokyo, Japan). The total cross-sectional skeletal muscle area (SMA) at the level of the third lumbar vertebra was calculated in the study population using Hounsfield unit thresholds of −29 to +150 for skeletal muscle [12]. The details of the measuring method are as described previously [13, 14]. In this study, the SMA (cm^2^) was divided by BSA (m^2^) to obtain the SMA/BSA index (SBI; cm^2^/m^2^), which is our original method of defining sarcopenia [14]. In the present study, patients with values less than the gender-specific lowest quartile of SBI were considered to have radiographic sarcopenia.

### Postoperative outcomes

For each patient, data regarding the following parameters were collected: postoperative mortality, morbidity including infectious complications, and postoperative length of hospital stay. Postoperative mortality included all in-hospital deaths before discharge. To analyze morbidity severity, each postoperative event was assessed and graded according to the Clavien–Dindo classification [15]. The major postoperative complications were defined as Clavien grade ≥ 3. POPF and delayed gastric emptying (DGE) were defined according to the International Study Group of Pancreatic Surgery guidelines [16, 17].

### Infectious complications

In the present study, we defined infectious complications as all postoperative infectious diseases including wound infections, infected abdominal fluid, intra-abdominal abscess, bacteremia, catheter-related infections, pneumonia, cholangitis, enteritis, and urinary tract infections. The definition of these infectious complications conformed to those of the American College of Surgeons National Surgical Quality Improvement Program criteria (NSQIP) [18]. Infected abdominal fluid was defined as drainage fluid with a positive culture from surgically replaced drains. Intra-abdominal abscess was defined as intra-abdominal fluid collection with positive cultures identified by ultrasonography or CT with clinical signs. A positive culture was not necessarily required in cases in which the NSQIP criteria were met and clinical signs were consistent with infectious complications.

### Risk factors for postoperative infections

Univariate and multivariate analyses were performed to identify the predictors closely related to infectious complications after PD among preoperative and intraoperative factors.

### Simple scoring system using perioperative risk factors

A simple scoring system was performed according to the results of multivariate analysis to investigate risk factors for postoperative infections. Patients were divided into three groups according to these risk factors, and the short-term outcomes of each group were then examined.

### Statistical analysis

JMP version 11 software (SAS Institute, Cary, NC) was used for all statistical analyses. Data were presented as means, medians, and standard deviations for continuous variables. Categorical data were presented as proportions. Differences between groups were assessed using the Mann-Whitney U-test for continuous variables, and Fisher’s exact test or chi-square test for categorical variables. To investigate the impact of prognostic factors associated with postoperative infections, we used a logistic regression model for univariate and multivariate analyses; odds ratios and 95% confidence intervals were calculated. A *P*–value <0.05 was considered statistically significant.

## Results

### Study population

Of the 241 patients who underwent PD, 22 were excluded because of the following reasons: unavailable preoperative CT images, 16; emergency surgery, 6. The demographic characteristics of the 219 patients (143 men [65.3%]; mean age, 65.9 years) are shown in Table 1. Pancreatic adenocarcinoma was the most common disease, occurring in 86 patients (39.3%). Preoperative biliary drainage was performed in 101 patients (46.1%). The mean operative time was 448 min (230–733 min) and the mean blood loss was 563 mL (10–3130 mL).

**Table 1:** Demographic and clinicopathological factors in patients with and without sarcopenia who underwent pancreaticoduodenectomy

	All patients (*n* = 219)	Non-sarcopenia (*n* = 164)	Sarcopenia (*n* = 55)	*P*-value
Demographic variables				
Sex (men)	143 (65.3)	107 (65.2)	36 (65.5)	0.98
Age (years)	65.9 (11.7)	65.1 (12.4)	68.2 (9.2)	0.18
BMI (kg/m^2^)	21.9 (3.3)	22.0 (3.4)	21.7 (3.0)	0.67
ASA physical status				
1–2/3	203/16	154/10	49/6	0.25
Laboratory value				
Albumin (g/dL)	4.0 (0.5)	4.1 (0.4)	3.9 (0.6)	0.006
Total lymphocyte (/mm^3^)	1642 (554)	1665 (561)	1572 (521)	0.30
Child–Pugh score				
A/B	205/14	155/9	50/5	0.36
Comorbidities				
Hypertension	89 (40.6)	68 (41.5)	21 (38.2)	0.67
Diabetes	64 (29.2)	43 (26.2)	21 (38.2)	0.10
Etiology of the disease				
Pancreatic adenocarcinoma	86 (39.3)	61 (37.2)	25 (45.5)	0.31
Bile duct carcinoma	27 (12.3)	20 (12.2)	7 (12.7)	
Ampullary adenocarcinoma	28 (12.8)	18 (11.0)	10 (18.2)	
Duodenal adenocarcinoma	10 (4.6)	9 (5.5)	1 (1.8)	
IPMN	32 (14.6)	26 (15.9)	6 (10.9)	
Others	36 (16.4)	30 (18.3)	6 (10.9)	
Preoperative biliary drainage	101 (46.1)	68 (41.5)	33 (60.0)	0.02
Intraoperative factor				
Operative time (min)	448 (93)	445 (93)	454 (94)	0.64
Blood loss (mL)	563 (486)	543 (443)	624 (596)	0.96
Vascular reconstruction	50 (22.8)	37 (22.6)	13 (23.6)	0.87
Soft pancreatic texture	133 (60.7)	101 (61.6)	32 (58.2)	0.66
Normal pancreatic duct (< 3 mm)	87 (39.7)	69 (42.1)	18 (32.7)	0.22

Data are presented as numbers (percentages) or means (± standard deviation).

*BMI* body mass index, *ASA* American Society of Anesthesiologists, *IPMN* intraductal papillary mucinous neoplasm

### Measurement of body composition

The mean SBI values were 74.7 ± 9.9 cm^2^/m^2^ for men and 58.3 ± 8.3 cm^2^/m^2^ for women. The mean SBI was significantly lower for women than for men (*P* < 0.001). The cut-off values for the lowest quartiles of SBI were 68.5 cm^2^/m^2^ for men and 52.5 cm^2^/m^2^ for women. Accordingly, 52 patients were categorized as having sarcopenia.

### Postoperative outcomes

The mortality and major complication rates for all 219 patients were 1.4% and 16.4%, respectively (Table 2). All three cases of mortality were caused by severe infectious complications. Of 219 patients, 103 (47.0%) had at least one postoperative infection. The most common infectious complications were infected abdominal fluid, 65; wound infections, 35; and intra-abdominal abscess, 12. The incidence rates of clinically relevant POPF (grade B or C) and DGE were 32.9% and 21.9%, respectively. The median length of hospital stay was 30 d (interquartile range, 24–40 d).

**Table 2:** Postoperative outcomes in patients with and without sarcopenia who underwent pancreaticoduodenectomy

	All patients (*n* = 219)	Non-sarcopenia (*n* = 164)	Sarcopenia (*n* = 55)	*P*-value
Mortality	3 (1.4)	0 (0)	3 (5.5)	0.004
Major complication	36 (16.4)	24 (14.6)	12 (21.8)	0.21
Any infectious complication	103 (47.0)	66 (40.2)	37 (67.3)	<0.001
Wound infection	35 (16.0)	26 (15.9)	9 (16.4)	0.93
Intra-abdominal abscess	12 (5.5)	6 (3.7)	6 (10.9)	0.04
infected abdominal fluid	65 (29.7)	41 (25.0)	24 (43.6)	0.009
Bacteremia	7 (3.2)	2 (1.2)	5 (9.1)	0.004
Catheter-related infection,	9 (4.1)	7 (4.3)	2 (3.6)	0.84
Pneumonia	4 (1.8)	1 (0.6)	3 (5.5)	0.02
Cholangitis	8 (3.7)	4 (2.4)	4 (7.3)	0.10
Enteritis	5 (2.3)	5 (3.1)	0 (0)	0.19
Urinary tract infection	2 (0.9)	1 (0.6)	1 (1.8)	0.42
Pancreatic fistula (Grade B + C)	72 (32.9)	52 (31.7)	20 (36.4)	0.52
Delayed gastric emptying	48 (21.9)	36 (22.0)	12 (21.8)	0.98
Length of stay, median (range)	30 (24–40)	30 (24–38)	30 (25–41)	0.25

Data are presented as numbers (percentages), unless otherwise indicated

### The impact of sarcopenia

The clinical demographic characteristics of patients with and without sarcopenia are shown in Table 1. Patients in the sarcopenia group had significantly lower albumin levels and a higher rate of preoperative biliary drainage, but other factors, including intraoperative factors, were not significantly different. Postoperative outcomes associated with the presence or absence of sarcopenia are presented in Table 2. The mortality rate was significantly higher in the sarcopenia group (5.5% vs. 0%, *P* = 0.004). Although the incidences of major complications, POPF, and DGE were not significantly different between the groups, the sarcopenia group had a significantly higher infectious complication rate (67.3% vs. 40.2%, *P* < 0.001). The length of postoperative hospital stay did not significantly differ between the groups.

### Comparison between patients with and without postoperative infections

Patient characteristics are shown in Table 3. There were no differences between patients with and without infections with respect to sex, age, BMI, ASA physical status, laboratory values, liver function, comorbidities, etiology of disease, blood loss, vascular reconstruction, and pancreatic duct diameter. Sarcopenia, preoperative biliary drainage, operative time, and soft pancreatic texture were more common (or longer, in the case of operative time) in patients with infections.

**Table 3:** Univariate and multivariate analyses of perioperative predictors associated with postoperative infections in patients who underwent pancreaticoduodenectomy

Variable			Univariate analysis			Multivariate analysis		
	Non-infection (*n* = 116)	Infection (*n* = 103)	OR	95% CI	*P*-value	OR	95% CI	*P*-value
Sarcopenia	18 (15.5)	37 (35.9)	3.05	1.62–5.91	<0.001	3.43	1.71–7.14	<0.001
Male	77 (66.4)	66 (64.1)	0.90	0.52–1.58	0.72			
Age (≥ 70 years)	45(38.8)	47 (45.6)	1.32	0.77–2.27	0.31			
BMI (≥ 25 kg/m^2^)	13 (11.2)	21 (20.4)	2.03	0.97–4.39	0.06	2.01	0.87–4.80	0.10
ASA (≥ grade 3)	6 (5.2)	10 (9.7)	1.97	0.70–5.98	0.20			
Albumin (< 3.6 g/dL)	14 (12.4)	19 (19.4)	1.70	0.81–3.66	0.16			
Child–Pugh score (grade B)	4 (3.4)	10 (9.7)	3.01	0.97–11.3	0.06	1.74	0.48–7.40	0.41
Diabetes	32 (27.6)	32 (31.1)	1.18	0.66–2.12	0.57			
Malignant disease	77 (66.4)	74 (71.8)	1.29	0.73–2.31	0.38			
Preoperative biliary drainage	42 (36.2)	59 (57.3)	2.36	1.38–4.09	0.002	2.20	1.17–4.20	0.014
Operative time (> 425 min)	60 (51.7)	69 (67.0)	1.89	1.10–3.30	0.02	1.57	0.83–2.99	0.17
Blood loss (> 550 mL)	39 (33.6)	46 (44.7)	1.59	0.92–2.76	0.09	1.92	1.00–3.73	0.048
Vascular reconstruction	28 (24.1)	23 (22.3)	0.95	0.50–1.78	0.87			
Soft pancreatic texture	59 (50.9)	74 (71.8)	2.47	1.41–4.37	0.001	3.71	1.96–7.29	<0.001
Normal pancreatic duct (< 3 mm)	42 (36.2)	45 (43.7)	1.37	0.79–2.36	0.26			

Data are presented as numbers (percentages).

*OR* odds ratio, *CI* confidence interval, *BMI* body mass index, *ASA* American Society of Anaesthesiologists

### Risk factors for postoperative infections

Table 3 shows the results of univariate and multivariate analyses used to identify the predictors closely related to infectious complications after PD. In univariate analysis, four variables (sarcopenia, preoperative biliary drainage, operative time, and soft pancreatic texture) were found to be significant risk factors. Multivariate analysis showed that sarcopenia (odds ratio = 3.43; *P* < 0.001), preoperative biliary drainage (odds ratio = 2.20; *P* = 0.014), blood loss (odds ratio = 1.92; *P* = 0.048), and soft pancreatic texture (odds ratio = 3.71; *P* < 0.001) were significant risk factors for infectious complications after PD.

### Simple scoring system using perioperative risk factors

According to the number of significant risk (R) factors (sarcopenia, preoperative biliary drainage, blood loss, and soft pancreatic texture) in multivariate analysis, patients were divided into three groups: R0/1 group (*n* = 91), R2 group (*n* = 89), and R3/4 group (*n* = 39).

Table 4 shows the short-term outcomes after PD determined using the risk-scoring system. The incidence rates of mortality and major complications were not different between the groups. However, the infectious complication rates after PD were 28.6% for the R0/1 group, 49.4% for the R2 group, and 84.6% for the R3/4 group (*P* < 0.001). In a logistic regression model, all differences between the R0/1 group and other groups were significant.

**Table 4:** Short-term outcomes after pancreaticoduodenectomy based on the results of the risk scoring system

No. of risk factors	Mortality	*P*-value	Major complication	*P*-value	Infectious complication	*P*-value	OR	95% CI	*P*-value
R0/1 group (*n* = 91)	0 (0)	0.20	11 (12.1)	0.17	26 (28.6)	<0.001	-	-	-
R2 group (*n* = 89)	2 (2.2)		15 (16.9)		44 (49.4)		2.44	1.33–4.57	0.004
R3/4 group (*n* = 39)	1 (2.6)		10 (25.6)		33 (84.6)		13.8	5.47–40.0	<0.001

Data are presented as numbers (percentages).

*OR* odds ratio, *CI* confidence interval

## Discussion

This retrospective study demonstrated that sarcopenia is an independent prognostic factor of infectious complications after PD. To the best of our knowledge, this is the first study to identify the prognostic significance of sarcopenia on postoperative infections following PD. Concerning surgical procedures, the standard surgical procedure has changed to subtotal stomach-preserving PD from 2007 at our institution. In addition, the reconstruction of gastrojejunostomy has changed to an antecolic route from 2007 [11]. However, major changes in surgical procedures have not been introduced after 2007, so the results of this study should be valid.

In the entire cohort, the mortality, major complication, and infectious complication rates after PD were 1.4%, 16.4%, and 47.0%, respectively. The results obtained from our institution were better than those reported in previous papers [1, 2, 19]. However, the median length of stay was 30 d in the present study, which was much longer than the mean value reported from studies performed in Western countries.

Further improvements to surgical procedures and perioperative management are needed in order to improve postoperative outcomes after PD.

Sarcopenia is a syndrome defined by progressive and generalized loss of skeletal muscle mass and strength that occurs with aging or secondary to diseases [20, 21]. In the present study, we used preoperative CT to evaluate sarcopenia. CT is considered to be an objective and precise method for assessing sarcopenia [22–24]. Regarding the definition of sarcopenia, we used SBI to evaluate skeletal muscle mass. We considered that SBI would more precisely evaluate skeletal muscle mass in patients with different physiques, and would be a superior index for evaluating skeletal muscle mass [14]. In the present study, demographic characteristics, including age and body parameters, were not significantly different between the two groups.

The effect of sarcopenia on postoperative complications, especially POPF, has been reported previously [5–8]. In the present study, the incidence rates of major complications and POPF were not significantly different between the two groups. This finding differs from the results of previous studies. However, the sarcopenia group had a significantly higher incidence of in-hospital mortality and infectious complications. All cases of mortality were also the result of severe infectious complications. Concerning infectious complications, the incidence rates of intra-abdominal abscess and infected abdominal fluid were significantly higher in the sarcopenia group. Sarcopenia might negatively affect the healing process at the pancreatic anastomosis [6].

Our multivariate analysis revealed that sarcopenia, preoperative biliary drainage, blood loss, and soft pancreatic texture were perioperative risk factors related to postoperative infections after PD. Preoperative biliary drainage has been reported to be a risk factor for postoperative infections following PD. [25] Operative factors and pancreatic factors such as blood loss and soft pancreatic texture have been reported to be associated with POPF after PD, which may also be related to postoperative infections [6]. However, this represents a new finding demonstrating the association between sarcopenia and infections complications after PD.

In this study, we established a simple and comprehensive scoring system that predicted postoperative infections after PD. Thirty-nine patients (17.8%) had three or four risk factors (the R3/4 group), and these patients had 84.6% of all postoperative infectious complications. Assessing and managing these risk factors may improve postoperative outcomes, especially in high-risk patients. Nutritional intervention combined with physical exercise appear to be effective for the management of sarcopenia [26, 27]. Furthermore, perioperative antibiotic strategies to prevent bile contamination could prevent infectious complications after PD. [28] Finally, the development of specialized surgical procedures and techniques might contribute to reducing the rate of infectious complications.

Despite the important findings reported in this study, several limitations should be discussed. First, this was a small, single-center, retrospective study. Because of this, there may be some selection bias with respect to the patients who underwent PD. Second, we did not evaluate functional muscle status in terms of grip strength, walking speed, or exhaustion because of the retrospective nature of the study. The evaluation of both muscle mass and muscle function has been recommended in the diagnosis of sarcopenia [21], and future studies will be required to assess not only muscle mass but also function in sarcopenia. Third, we used the SBI for evaluating sarcopenia, which would be a useful modality for assessing sarcopenia. However, further studies are needed to assess the efficiency of SBI to diagnosis sarcopenia. Fourth, it remains unclear what nutritional interventions and physical exercise regimens would be valid for patients undergoing PD, because few studies have dealt with the effects of such interventions on sarcopenic patients with PD. Future studies are needed to examine the effects of perioperative interventions focusing on sarcopenia. Finally, there is insufficient evidence in the pathophysiology concerning the interaction between sarcopenia and infections. The depletion of skeletal muscle as a secretory organ of cytokines and peptides, and increasing adipose tissue as a key component of the immune system lead to the synthesis and secretion of several proinflammatory adipocytokines [29]. Thereby, decreased interleukin-15 and increased adipokines levels might be associated with the interaction between sarcopenia and immune depression [30]. Accordingly, We hypothesize that sarcopenia reflects the patients’ frailty, including impaired immune function, which ultimately leads to infections [14]. However, further research should investigate the molecular mechanism of sarcopenia’s effect on outcomes.

## Conclusions

In conclusion, the results of the present study indicate that sarcopenia is an objective and independent preoperative predictor of infectious complications after PD. Furthermore, assessing sarcopenia is easy and practicable. Accordingly, we propose that clinical assessment combined with sarcopenia may help clinicians to understand the risk of postoperative outcomes and determine perioperative management strategies.

## Abbreviations

ASA: American Society of Anesthesiologists
BMI: Body mass index
BSA: Body surface area
CT: Computed tomography
DGE: Delayed gastric emptying
NSQIP: National Surgical Quality improvement Program criteria
PD: Pancreaticoduodenectomy
POPF: Postoperative pancreatic fistula
SMA: Skeletal muscle area

## References

[1] Yoshioka R, Yasunaga H, Hasegawa K, Horiguchi H, Fushimi K, Aoki T (2014). Impact of hospital volume on hospital mortality, length of stay and total costs after pancreaticoduodenectomy. Br J Surg.

[2] Kimura W, Miyata H, Gotoh M, Hirai I, Kenjo A, Kitagawa Y (2014). A pancreaticoduodenectomy risk model derived from 8575 cases from a national single-race population (Japanese) using a web-based data entry system: the 30-day and in-hospital mortality rates for pancreaticoduodenectomy. Ann Surg.

[3] Okano K, Hirao T, Unno M, Fujii T, Yoshitomi H, Suzuki S (2015). Postoperative infectious complications after pancreatic resection. Br J Surg.

[4] Levolger S, van Vugt JL, de Bruin RW, IJzermans JN (2015). Systematic review of sarcopenia in patients operated on for gastrointestinal and hepatopancreatobiliary malignancies. Br J Surg.

[5] Sur MD, Namm JP, Hemmerich JA, Buschmann MM, Roggin KK, Dale W (2015). Radiographic Sarcopenia and Self-reported Exhaustion Independently Predict NSQIP Serious Complications After Pancreaticoduodenectomy in Older Adults. Ann Surg Oncol.

[6] Kirihara Y, Takahashi N, Hashimoto Y, Sclabas GM, Khan S, Moriya T (2013). Prediction of pancreatic anastomotic failure after pancreatoduodenectomy: the use of preoperative, quantitative computed tomography to measure remnant pancreatic volume and body composition. Ann Surg.

[7] Pecorelli N, Carrara G, De Cobelli F, Cristel G, Damascelli A, Balzano G (2016). Effect of sarcopenia and visceral obesity on mortality and pancreatic fistula following pancreatic cancer surgery. Br J Surg.

[8] Nishida Y, Kato Y, Kudo M, Aizawa H, Okubo S, Takahashi D (2016). Preoperative Sarcopenia Strongly Influences the Risk of Postoperative Pancreatic Fistula Formation After Pancreaticoduodenectomy. J Gastrointest Surg.

[9] Child CG (1944). Pancreaticojejunostomy and Other Problems Associated With the Surgical Management of Carcinoma Involving the Head of the Pancreas: Report of Five Additional Cases of Radical Pancreaticoduodenectomy. Ann Surg.

[10] Matsuda H, Sadamori H, Umeda Y, Shinoura S, Yoshida R, Satoh D (2012). Preventive effect of omental flap in pancreaticoduodenectomy against postoperative pseudoaneurysm formation. Hepato-Gastroenterology.

[11] Takagi K, Yagi T, Yoshida R, Shinoura S, Umeda Y, Nobuoka D (2016). Surgical Outcome of Patients Undergoing Pancreaticoduodenectomy: Analysis of a 17–Year Experience at a Single Center. Acta Med Okayama.

[12] Mitsiopoulos N, Baumgartner RN, Heymsfield SB, Lyons W, Gallagher D, Ross R (1998). Cadaver validation of skeletal muscle measurement by magnetic resonance imaging and computerized tomography. J Appl Physiol (1985).

[13] Takagi K, Yagi T, Yoshida R, Shinoura S, Umeda Y, Nobuoka D (2016). Sarcopenia and American Society of Anesthesiologists Physical Status in the Assessment of Outcomes of Hepatocellular Carcinoma Patients Undergoing Hepatectomy. Acta Med Okayama.

[14] Takagi K, Yagi T, Yoshida R, Umeda Y, Nobuoka N, Kuise T (2017). Sarcopenia predicts postoperative infection in patients undergoing hepato-biliary-pancreatic surgery. Int J Surg Open.

[15] Clavien PA, Barkun J, de Oliveira ML, Vauthey JN, Dindo D, Schulick RD (2009). The Clavien-Dindo classification of surgical complications: five-year experience. Ann Surg.

[16] Bassi C, Dervenis C, Butturini G, Fingerhut A, Yeo C, Izbicki J (2005). Postoperative pancreatic fistula: an international study group (ISGPF) definition. Surgery.

[17] Wente MN, Bassi C, Dervenis C, Fingerhut A, Gouma DJ, Izbicki JR (2007). Delayed gastric emptying (DGE) after pancreatic surgery: a suggested definition by the International Study Group of Pancreatic Surgery (ISGPS). Surgery.

[18] Bilimoria KY, Liu Y, Paruch JL, Zhou L, Kmiecik TE, Ko CY (2013). Development and evaluation of the universal ACS NSQIP surgical risk calculator: a decision aid and informed consent tool for patients and surgeons. J Am Coll Surg.

[19] Pecorelli N, Balzano G, Capretti G, Zerbi A, Di Carlo V, Braga M (2012). Effect of surgeon volume on outcome following pancreaticoduodenectomy in a high-volume hospital. J Gastrointest Surg.

[20] Morley JE, Baumgartner RN, Roubenoff R, Mayer J, Nair KS (2001). Sarcopenia. J Lab Clin Med.

[21] Cruz-Jentoft AJ, Baeyens JP, Bauer JM, Boirie Y, Cederholm T, Landi F (2010). Sarcopenia: European consensus on definition and diagnosis: Report of the European Working Group on Sarcopenia in Older People. Age Ageing.

[22] Rockwood K, Stadnyk K, MacKnight C, McDowell I, Hébert R, Hogan DB (1999). A brief clinical instrument to classify frailty in elderly people. Lancet.

[23] Fried LP, Tangen CM, Walston J, Newman AB, Hirsch C, Gottdiener J (2001). Frailty in older adults: evidence for a phenotype. J Gerontol A Biol Sci Med Sci.

[24] Rockwood K, Song X, MacKnight C, Bergman H, Hogan DB, McDowell I (2005). A global clinical measure of fitness and frailty in elderly people. CMAJ.

[25] Chen Y, Ou G, Lian G, Luo H, Huang K, Huang Y (2015). Effect of Preoperative Biliary Drainage on Complications Following Pancreatoduodenectomy: A Meta-Analysis. Medicine (Baltimore).

[26] Calvani R, Miccheli A, Landi F, Bossola M, Cesari M, Leeuwenburgh C (2013). Current nutritional recommendations and novel dietary strategies to manage sarcopenia. J Frailty Aging.

[27] Denison HJ, Cooper C, Sayer AA, Robinson SM (2015). Prevention and optimal management of sarcopenia: a review of combined exercise and nutrition interventions to improve muscle outcomes in older people. Clin Interv Aging.

[28] Sudo T, Murakami Y, Uemura K, Hashimoto Y, Kondo N, Nakagawa N (2014). Perioperative antibiotics covering bile contamination prevent abdominal infectious complications after pancreatoduodenectomy in patients with preoperative biliary drainage. World J Surg.

[29] Tilg H, Moschen AR (2006). Adipocytokines: mediators linking adipose tissue, inflammation and immunity. Nat Rev Immunol.

[30] Lutz CT, Quinn LS (2012). Sarcopenia, obesity, and natural killer cell immune senescence in aging: altered cytokine levels as a common mechanism. Aging (Albany NY).

